# Automatic OptoDrive for Extracellular Recordings and Optogenetic Stimulation in Freely Moving Mice

**DOI:** 10.1523/ENEURO.0015-25.2025

**Published:** 2025-06-18

**Authors:** Alberto Caballero-Ruiz, Erick Lopez-Roldan, Monica Luna, Luis Rodriguez-Blanco, Leopoldo Emmanuel Polo-Castillo, Mario G. Moreno, Leopoldo Ruiz-Huerta, Ranier Gutierrez

**Affiliations:** ^1^Instituto de Ciencias Aplicadas y Tecnología (ICAT), Universidad Nacional Autónoma de México (UNAM), Circuito Exterior S/N, Ciudad Universitaria, Coyoacán, Mexico City, C.P. 04510, Mexico; ^2^National Laboratory for Additive and Digital Manufacturing (MADiT), Mexico City, C.P. 04510, Mexico; ^3^Programa de Maestría y Doctorado en Ingeniería, Universidad Nacional Autónoma de México (UNAM), Mexico City 04510, Mexico; ^4^Laboratory Neurobiology of Appetite; Department of Pharmacology, Cinvestav, CDMX 07360, Mexico; ^5^Laboratory Neurobiology of Appetite, Centro de Investigación sobre el Envejecimiento (CIE), Cinvestav sede sur, CDMX 14330, México

**Keywords:** freely moving mice, multichannel recordings, reimplantable electrode arrays

## Abstract

Extracellular recordings in freely moving mice, especially those with movable electrodes (microdrives), are crucial for understanding brain function. However, existing microdrives are often heavy, expensive, fragile, and unsuited for long-term studies with multichannel recordings. The OptoDrive is a new, lightweight (3.2 g), low-cost system for chronic neural recordings and optogenetic manipulation in mice. It features a detachable, 16-channel tungsten-wire electrode assembly with a 3 mm stroke (15 μm step displacement) and an integrated optical fiber. This system enables repeated implantation and explantation without surgery, requiring only gas anesthesia. The OptoDrive has demonstrated stable recordings from the lateral hypothalamus of freely behaving mice for nearly 1 month and successful optogenetic silencing of neuronal activity. In conclusion, OptoDrive offers a cost-effective, compact solution for long-term electrophysiology and optogenetics in freely moving mice.

## Significance Statement

The OptoDrive is a motorized microdrive system for long-term neural recording and optogenetic stimulation in freely behaving rodents. It uses a miniature linear actuator with a stepper micromotor and additive-manufactured parts for precise 15 μm resolution manipulation of a 16-tungsten-wire array with a 3 mm stroke and can include fiber optics for optogenetic stimulation. To address the challenges of cost, weight, reusability, and long-term recording, OptoDrive offers an affordable, efficient, and easily assembled solution for chronic neuroscience experiments, enhancing the study of neural activity in mice.

## Introduction

To understand neural circuits in behaving animals, precise tools capable of simultaneous neural monitoring and brain stimulation are needed. A vital contribution to the advancement of knowledge regarding brain function has been made by traditional recording methods employed in freely moving rodents. An example of such methods is provided by fixed-implant systems, through which single- or multi-unit recordings could be obtained (Nicolelis et al., 1997) to manually movable systems (Morrow, 1980; Korshunov, 2006; Chang et al., 2013; Osanai et al., 2019) or motorized systems based on different types of actuators, such as CD brushless motors (Fee and Leonardo, 2001; Yamamoto and Wilson, 2008), hydraulic systems (Sato et al., 2007), or piezoelectric actuators (Yang et al., 2011; Polo-Castillo et al., 2019; Smith et al., 2022). However, the application of these techniques often faces challenges concerning weight, scalability, and flexibility, whereby their utility for the investigation of large-scale neural dynamics during natural behaviors, particularly in mice, is constrained. While many neurons are recorded by advanced silicon probes such as Neuropixels, these probes are predominantly designed for head-fixed setups, although exceptions exist (Juavinett et al., 2019; van Daal et al., 2021). The widespread adoption of Neuropixels systems is limited by their high cost, and their reuse in the same animal is rendered unlikely by their fragility.

Optogenetics has revolutionized neuroscience, enhancing precise manipulation of neural activity. This technique uses light to stimulate genetically modified neurons (Anikeeva et al., 2012; Freedman et al., 2016; Prado et al., 2016; Bimbard et al., 2025). A key advancement was pioneered by Anikeeva et al. (2012) through the integration of a fiber optic with an electrode microdrive, and the term “optrode” was coined. In this initial manual design, an optrode was featured for the light stimulation of genetically modified neurons, and their electrical activity was simultaneously recorded. A fixed optrode for recording and optogenetic stimulation in freely moving mice was previously developed (Prado et al., 2016).

A previous study introduced a low-cost, lightweight microdrive for long-term chronic recording and reimplantation in freely behaving rats (Polo-Castillo et al., 2019). The previous microdrive, despite being low-cost and suitable for long-term recordings and reimplantation in rats, was too heavy for mice and lacked optogenetic capabilities. The present work introduces OptoDrive, a novel microdrive designed for mice. OptoDrive retains the prior advantages (low cost, long-term chronic recordings, and reimplantation) but significantly reduces weight for mice compatibility and incorporates optogenetic stimulation, enabling simultaneous recording and manipulation of neural activity in freely behaving mice.

## Materials and Methods

### OptoDrive design

The proposed automated microdisplacement system was designed for performing extracellular recording and/or light stimulation in rodents (rats and mice); hence, its weight must be <3.5 g. Such a system must be able to move an array of 16 microelectrodes along a stroke of 3 mm with micrometric resolution and ensure smooth and stable displacement.

**Figure 1.**
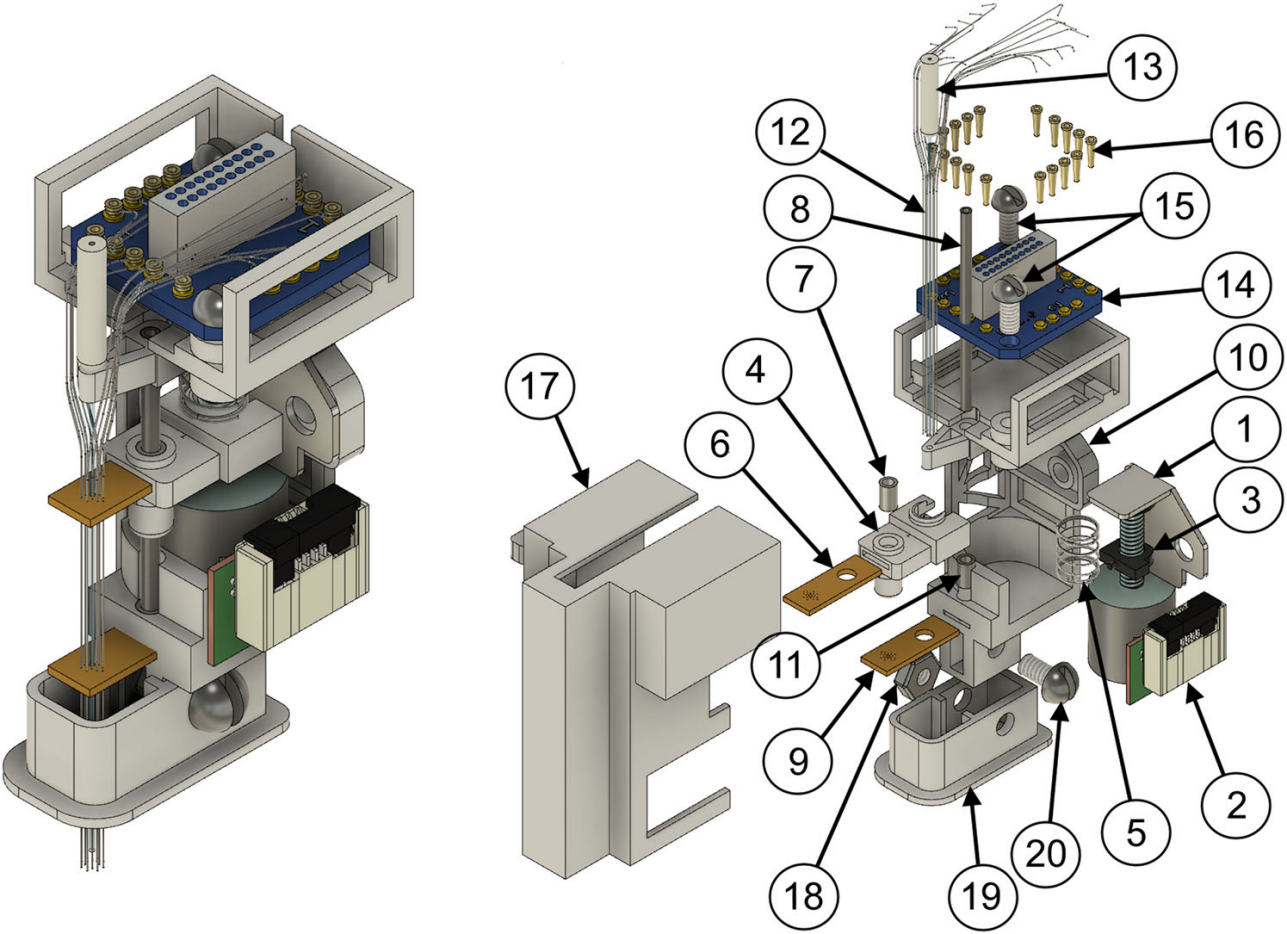
The proposed design and exploded view of the automated microdisplacement system (OptoDrive). (1) Miniature linear actuator, (2) 4p FPC/FFC connector, (3) nut of the miniature linear actuator, (4) electrode shuttle, (5) compression spring, (6) upper support board, (7) upper bushing, (8) guide, (9) lower support board, (10) OptoDrive main body, (11) lower bushing, (12) microelectrodes, (13) optical fiber with its ferrule, (14) gold-plated electronic interface board, (15) screws, (16) gold plate pins, (17) body cover, (18) fixing nut, (19) OptoDrive baseplate, (20) fixing screw.

The OptoDrive design and its exploded view are presented in Figure 1. A miniature linear actuator constitutes its core (1) based on a stepper micromotor, in which a PCB (2) with a 4p FPC/FFC connector for stable electrical connections is incorporated. Motion is transmitted to the electrode shuttle (4) by a nut (3), while preload is applied by a compression spring (5), whereby backlash is minimized and precise vertical displacement is ensured. By means of upper (6) and lower (9) support boards, through the utilization of bushings (7, 11) and a guide (8), the aligned linear motion of the microelectrodes is ensured. This assembly is housed within the main body (10), by which two configurations are made possible: 16-microelectrode extracellular recording only (12) or combined recording and optogenetic stimulation via an optical fiber with a ferrule (13). The microelectrodes (12) are connected to a 16/18 channel headstage via gold pins (16) by a gold-plated electronic interface board (EIB) (14), which is secured with screws (15). The assembly is protected by a body cover (17). The OptoDrive is affixed to a skull-mounted baseplate (19) using a nut (18) and a screw (20). This baseplate is the sole permanently fixed component [secured with Metabond to the skull, after which Super Glue (Loctite) is applied around its perimeter], access to common recording sites is permitted, and device replacement is facilitated.

### Fabrication and assembly

**Figure 2.**
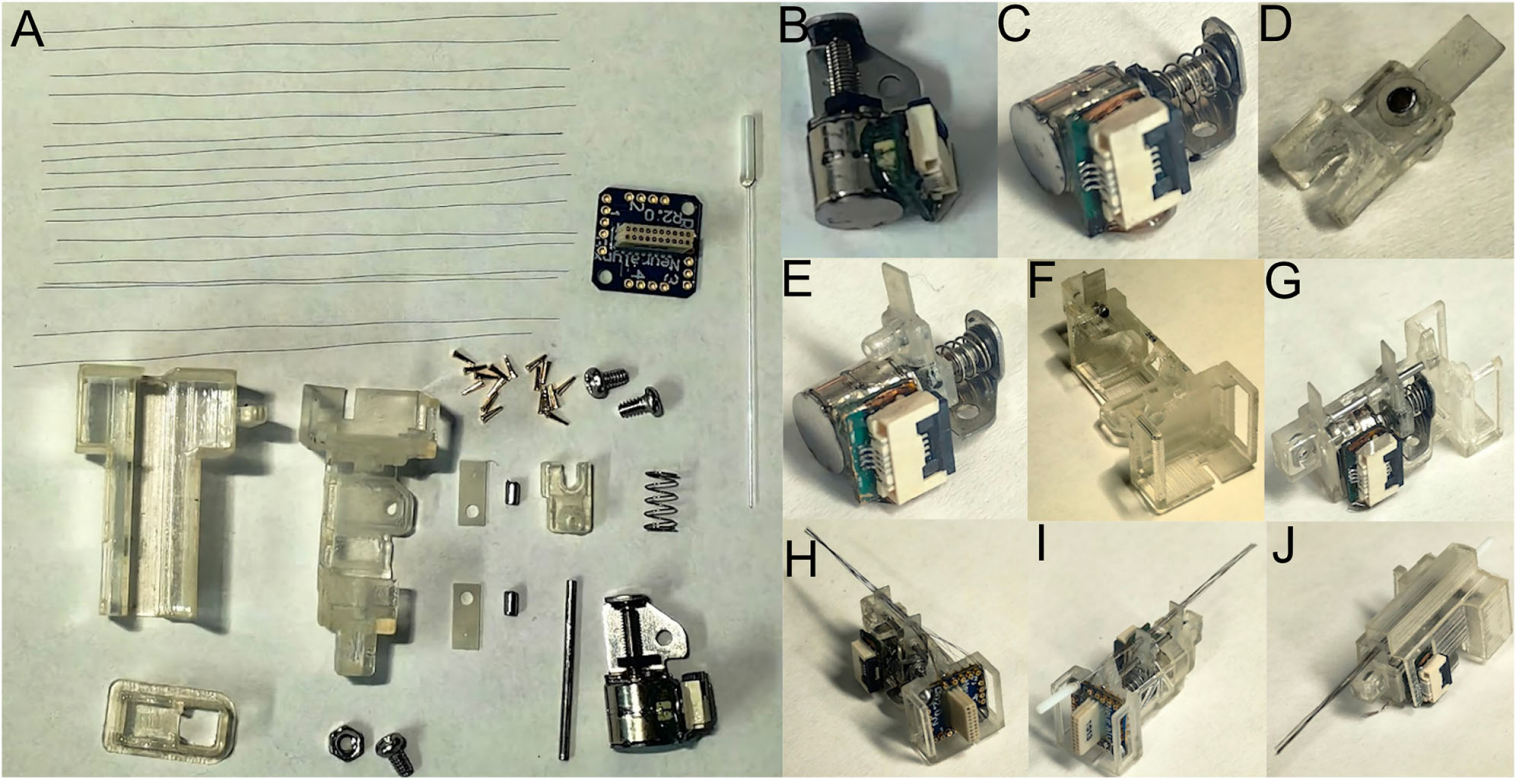
OptoDrive assembly process. ***A***, OptoDrive components; ***B***, miniature linear actuator and 4p FPC/FFC assembly; ***C***, spring and miniature linear actuator assembly; ***D***, electrode shuttle and upper support board assembly, fixed by the upper bushing; ***E***, assembly of sections ***C*** and ***D***; ***F***, lower support board and main OptoDrive body assembly via the lower bushing; ***G***, assembly of sections ***E*** and ***F*** with the guide; ***H***, assembly of the EIB-16 using two screws and attachment of the electrodes to the EIB-16 board using gold-plated pins; ***I***, assembly of the optical fiber; and ***J***, assembly of the body cover. For the bill of materials, see Extended Data Figure 2-1, and for the development files, see Gutierrez and Caballero-Ruiz (2025) (https://doi.org/10.17605/OSF.IO/DKR42).

The OptoDrive's 16 components (Fig. 2*A*) include four additively manufactured, five CNC/custom-tooled, one PCB, and eight commercial items (Extended Data Fig. 2-1 for a detailed bill of materials). The four additively manufactured parts (main body, electrode shuttle, body cover, and baseplate, with a combined weight of ∼1.3 g, manufactured with material jetting technology, e.g., Stratasys Connex 3 with RGD720 material, or similar precision technology) were designed for minimal weight, precise tolerances, and screwless assembly of the displacement mechanism. The CNC/custom-fabricated components include two support boards (configurable for recording only or with a central 0.28 mm hole for an optical fiber for optogenetics), a guide (cut from a G20 needle), and two bushings (cut from G18 needles). A lab-designed PCB incorporates a 4p 0.5 mm pitch FPC/FFC connector. The key commercial components comprise a miniature linear actuator (1.17 g; with a stepper micromotor providing 15 µm full-step resolution), an EIB-16 (NeuraLynx) with gold plate pins, 17 tungsten microwires (Ø35 µm, formvar insulated; California Fine Wire), and an optional optical fiber with a ferrule (Thorlabs FT200UMT; 0.39 NA, Ø200 µm core). Standard items such as the compression spring and M1.6 × 0.34 fasteners are also utilized.

10.1523/ENEURO.0015-25.2025.f2-1 Figure 2-1 Optodrive Bill of Materials. Download Figure 2-1, DOCX file.

The complete system, including the baseplate, fixation nut, and screw, weighs ∼3.4 g and has overall dimensions of 15 × 15 × 22 mm in both configurations. Figure 2*A* shows the OptoDrive components. The OptoDrive assembly (Fig. 2*B–J*) proceeds through the following steps: the 4p FPC/FFC connector is soldered to the PCB, which is then connected to the miniature linear actuator (Fig. 2*B*). The preloading spring is inserted between the upper face of the miniature linear actuator and the lead screw nut (Fig. 2*C*). Using the guide, the upper support board is fixed to the electrode shuttle via the upper bushing (Fig. 2*D*); this subassembly is subsequently inserted into the leadscrew nut (Fig. 2*E*). Within the main body, the lower support board is inserted and fixed via the lower bushing with the aid of the guide (Fig. 2*F*). The miniature linear actuator is then inserted into the main body, along with the guide, to align all the displacement system components (Fig. 2*G*). The EIB-16 is affixed to the upper part of the main body with two screws, and the fixing nut is inserted into the lower part. The electrode shuttle is moved to its closest position relative to the lower support board to facilitate electrode assembly. The electrodes are inserted through the support board orifices and connected to the EIB-16 via gold-plated pins (Fig. 2*H*), handling them carefully to prevent bending. UV light-curing glue is applied to the upper support board to secure the electrodes to the shuttle. If optogenetic stimulation is needed, the optical fiber is inserted prior to UV glue application. The fiber is coated with white petrolatum to prevent its fixation to the upper support board and to maintain its position (Fig. 2*I*). Finally, the body cover is installed (Fig. 2*J*). The assembly task took ∼30 min. As depicted in Figure 2*I*, the electrode array is not housed within a polyimide tube or moved through a metal cannula. Instead, the micromechanical support provided by the upper and lower support boards ensures that the electrodes resist bending upon insertion into the brain and maintain their 4 × 4 array configuration.

### OptoDrive control system

The positioning system was driven by an A4988 stepper motor driver under the control of an Arduino Uno R3 microcontroller. The OptoDrive's movement was software programmed, allowing forward and reverse displacements in 60 μm increments. Two push buttons initiate these increments. This step size is appropriate for searching for distinct neural populations, but the system's resolution can be modified through software adjustments to the mechanical resolution of 15 μm increments [circuit diagrams and program codes are provided in Gutierrez and Caballero-Ruiz (2025); https://doi.org/10.17605/OSF.IO/DKR42].

### Experimental model and subject details

An adult male C57BL/6 mouse [for targeting the subcortical areas of the zona incerta (ZI), lateral hypothalamus area (LHA), tuberal nucleus (TU)] and one Vglut2-IRES-Cre mouse (for optogenetic manipulation of LHA glutamatergic neurons) were used. The mice were individually housed at 22 ± 1°C on a 12 h light/dark cycle with *ad libitum* access to chow. All procedures were approved by the CINVESTAV Animal Care and Use Committee (CICUAL).

### Stereotaxic surgeries

The mice were anesthetized with 1.5% isoflurane and placed in a stereotaxic instrument (Stoelting). Ophthalmic ointment was used to maintain eye lubrication (hydrocortisone, neomycin, and polymyxin B). Lidocaine (0.05 ml, subcutaneous) was applied to the incision site for analgesia, and enrofloxacin (0.4 ml/kg) was injected for 3 d after surgery as an antibiotic. The OptoDrive electrodes were stained with CellTracker CM-Dil (C7001, Invitrogen) and implanted into the ZI (−1.3 mm posterior to bregma, ±1.0 mm lateral to midline, and −4.9 mm ventral to the skull surface). The implant was secured with one layer of adhesive cement (C&B Metabond; Parkell), followed by acrylic (Nic Tone, MDC Dental). The electrode tracks were confirmed by preparing coronal sections (40 µm, with a cryostat, Thermo Scientific) and observing them with an epifluorescence microscope (Nikon, Eclipse E200). The mouse was again anesthetized with 1.5% isoflurane for the new implantation procedure and placed into a stereotaxic instrument. The OptoDrive was detached from the baseplate by unscrewing and effortlessly extracting it. The baseplate region was cleansed with saline solution, encompassing the trepan, whereupon a fresh OptoDrive was implanted.

### Recording sessions with only an electrode array

The mice were habituated to a 30 × 20 × 20 cm acrylic chamber. For freely behaving recordings, a rubber band minimized cable drag and facilitated mouse movement during recordings. Each session began with a 10 min single-unit recording to establish a baseline. Two additional 10 min recording sessions were subsequently conducted, with a 60 µm micromovement performed between them. This protocol resulted in a total session duration of ∼30 min. Three consecutive sessions were conducted, involving a displacement (ascending or descending) of 0.6 mm, each contributing 0.2 mm to the total. After these sessions, there was a 48 h interval before the first session, with displacement in the opposite direction.

Afterward, a new OptoDrive was implanted, and 1 week after the new implantation, another set of sessions involving displacement was carried out. The recordings aimed at exploring neural activity as the OptoDrive moved either upward or downward through three distinct brain regions: the ZI, LHA, and TU.

Neural activity was amplified and digitized by a 64-channel PZ5 NeuroDigitizer and processed by an RZ2 BioAmp Processor (Tucker-Davis Technologies). The signals were sampled at 50 KHz (28 bit resolution), bandpass filtered (0.5–8 KHz), and action potentials were identified online via voltage–time threshold windows. Single units required amplitudes >50 µV and spikes 3.5–4 standard deviations above the channel's mean signal. The spikes were exported via OpenBridge software (Tucker-Davis Technologies) and sorted via a Plexon Offline Sorter. Only single units with stable waveforms throughout the session were analyzed (Gutierrez et al., 2010).

### Recording sessions with optical fiber

A 473 nm DPSS laser (OEM laser), synchronized with Med Associates software and a TTL generator, delivered light via a 200 μm core and 0.39 NA multimode fiber (Thorlabs FT200UMT). The patch cord power, set to 15 mW (Thorlabs PM20A), yielded 10–12.6 mW at the fiber tip. Adult Vglut2-IRES-Cre mice were targeted at the LHA (AP:−1.3, ML:1.1, DV:−5.2). They underwent a 30 min scanner laser frequency task (Prado et al., 2016), receiving 2 s of random stimulation (frequencies: control; 5, 10, 20, 33, 40, or 50 Hz with 10 ms pulses; or 2 s continuous), followed by 4 s off.

### Histology and microscopy

The mice were deeply anesthetized with pentobarbital (150 mg/kg) and intracardially perfused with PBS, pH 7.4, and then with 4% paraformaldehyde (PFA). Heads were fixed in PFA (4°C, 1 d), and the brains were removed and cryoprotected in 30% sucrose with sodium azide (4°C, 3 d). Coronal sections (40 µm), cut using a cryostat (Thermo Scientific HM525 NX), were imaged with a Gryphax camera on a Nikon Eclipse E200 epifluorescence microscope (4× objective).

### Data analysis

Custom-made MATLAB (R2023a, MathWorks) code was used to plot single-unit activity throughout the sessions.

## Results

The experiments focused on two areas, mechanical and application performance, including stable recordings, neuronal yield, and optostimulation, to evaluate the optimal performance.

### OptoDrive mechanical assessment

**Figure 3.**
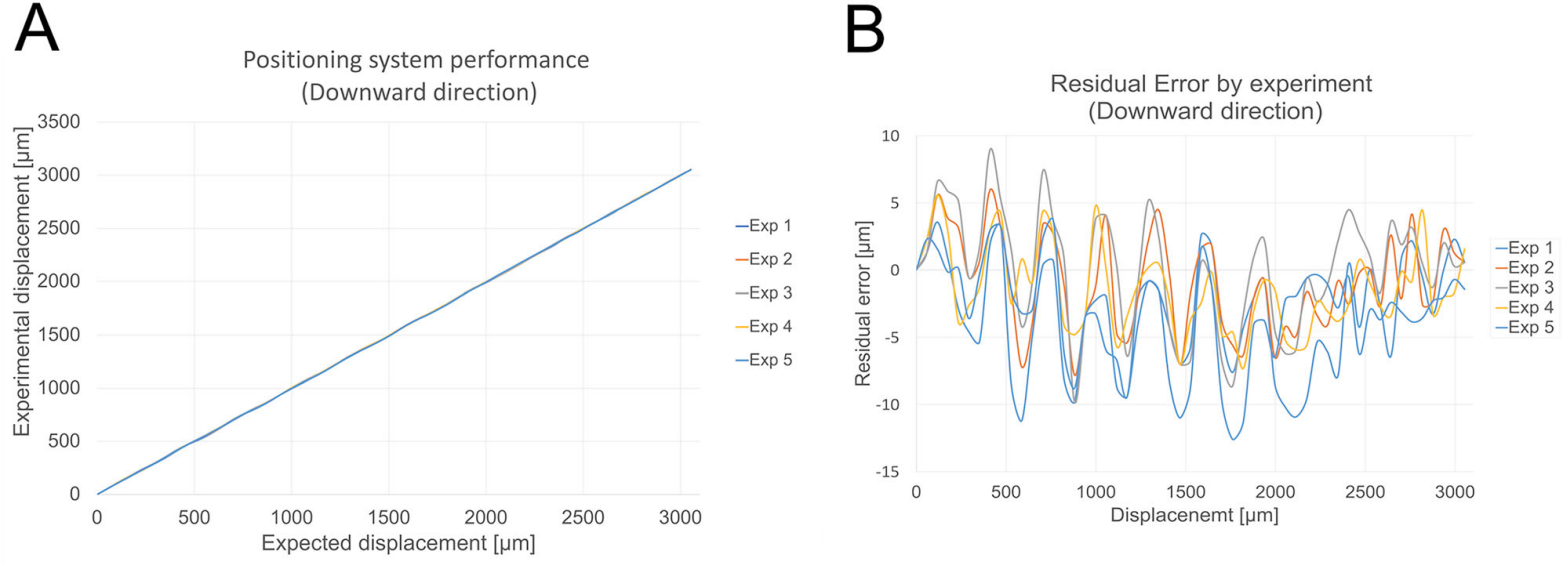
Accuracy of the electrode positioning system during downward movement. ***A***, Experimental position versus expected position. ***B***, Residual error for each displacement experiment.

The OptoDrive system (including baseplate, nut, and screw) weighs ∼3.4 g, measures 15 × 15 × 22 mm, and offers a 3 mm travel range with 15 μm resolution, which is suitable for rodent brain studies. The displacement quality assessment (Movie 1) confirmed smooth electrode motion without vibrations or lateral movements, which is crucial for reliable recording. Positioning error, assessed via a NIKON Profile Projector V-16D (1 μm resolution), involved 50 programmed 60 μm incremental displacements over the 3 mm range (five experiments each, downward and upward). The mean achieved displacements were 58.72 μm (downward) and 58.75 μm (upward), with standard deviations of 2.76 and 2.39 μm, respectively. Figure 3, which details the downward movement performance, shows nearly identical experimental trajectories (Fig. 3*A*). The residual errors (Fig. 3*B*), which are based on the mean step displacement, ranged from −12.6 μm to +8.89 μm (21.49 μm total range), with a standard deviation of 3.98 μm.

**Movie 1.** OptoDrive moving up and down. [View online]

### Recordings with only electrodes from freely moving mice

#### First implant

**Figure 4.**
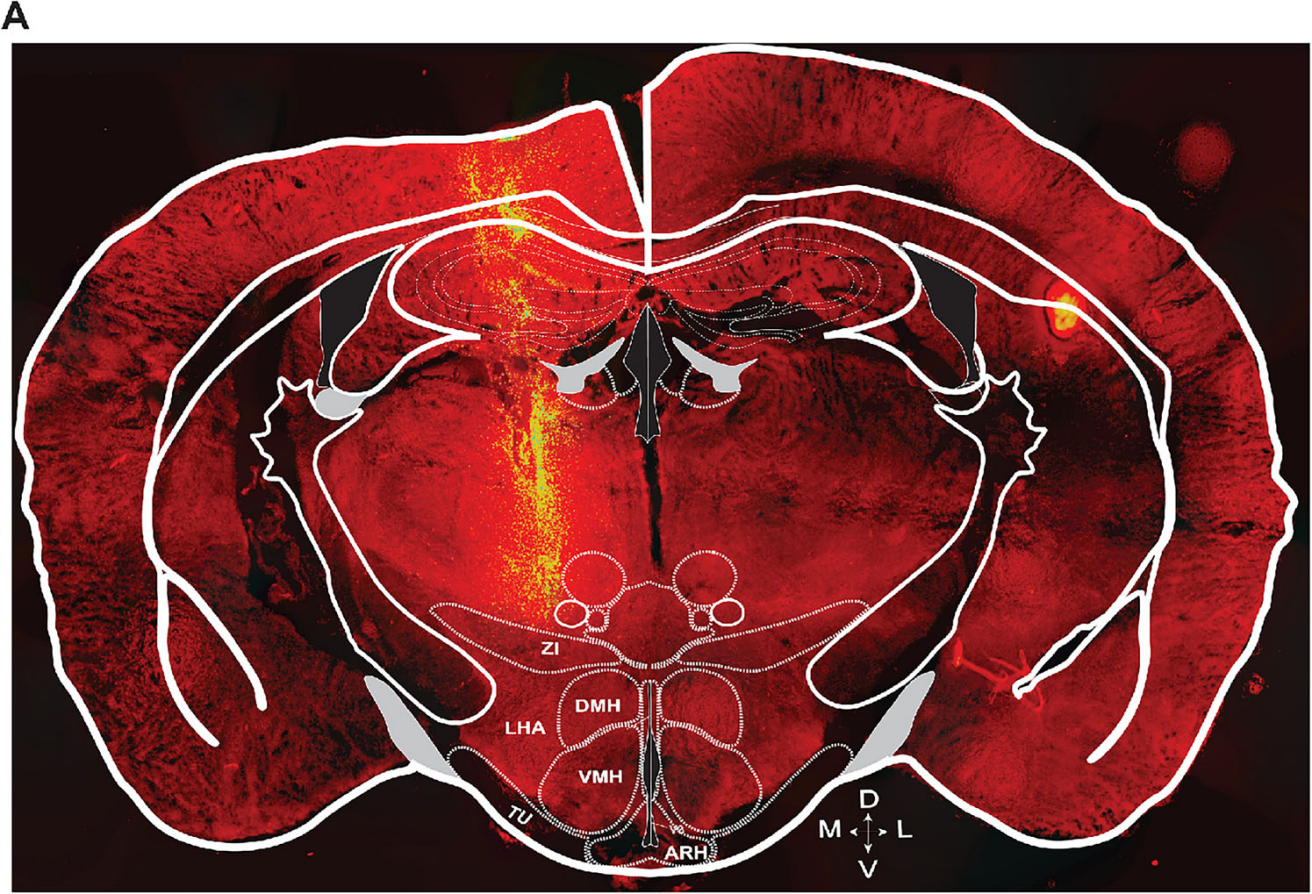
Trace of OptoDrive (only electrodes) on a coronal mouse brain section. ***A***, A 40 µm-thick coronal section 4× image montage of the OptoDrive stained with CellTracker CM-Dil. The following abbreviations are used: ARH, arcuate nucleus; DMH, dorsomedial hypothalamus; LHA, lateral hypothalamic area; VMH, ventromedial hypothalamus; TU, tuberal nucleus; ZI, zona incerta.

In vivo recordings were conducted in one adult male C57BL/6 wild-type mouse to evaluate OptoDrive functionality. Behavioral observations confirmed that the mouse exhibited normal locomotion and exploratory behavior when they were implanted with OptoDrive (Movie 2). Over 10 recording sessions, we performed 16 discrete electrode adjustments, yielding 162 well-isolated single units in total from one mouse. These units were localized to three distinct brain regions: the ZI, LHA, and TU. Figure 4 shows the initial electrode track, visualized with CellTracker CM-DiI, extending to the ZI. This represents the electrode's position immediately following the postsurgical recovery period (note that subsequent electrode movements are not visible with this method owing to the dye's temporal characteristics). Following a 1 week postsurgical recovery period, preliminary experiments (data not shown) involved a total electrode descent of 0.6 mm. The data presented here represent the first formal experiment following these initial tests, with the electrode positioned within the TU.

**Movie 2.** Mice moving with the OptoDrive. [View online]

#### Ascending movements of the OptoDrive

The following protocol was implemented to evaluate the recording stability, neuronal yield, and functional performance of the OptoDrive. On each recording day, a baseline 10 min recording of spontaneous neural activity was obtained from a freely moving mouse. Crucially, the electrodes’ position remained fixed during this baseline period. Following the baseline, the mouse was anesthetized, and the electrodes were advanced (or retracted) by a single 6 µm increment using the integrated micromotor. A second 10 min recording was then initiated. This process was repeated, with the mouse being anesthetized and the electrodes moving another 60 µm, followed by a third 10 min recording. This procedure resulted in three 10 min recording sessions per day, each corresponding to a distinct position of the electrodes. This protocol allowed us to consider each 10 min recording as an independent data point, enabling within-day comparisons (immediately following electrode movement) and between-day comparisons (at 24, 48 h, or 1 week intervals with no electrode movement) of recording stability.

**Figure 5.**
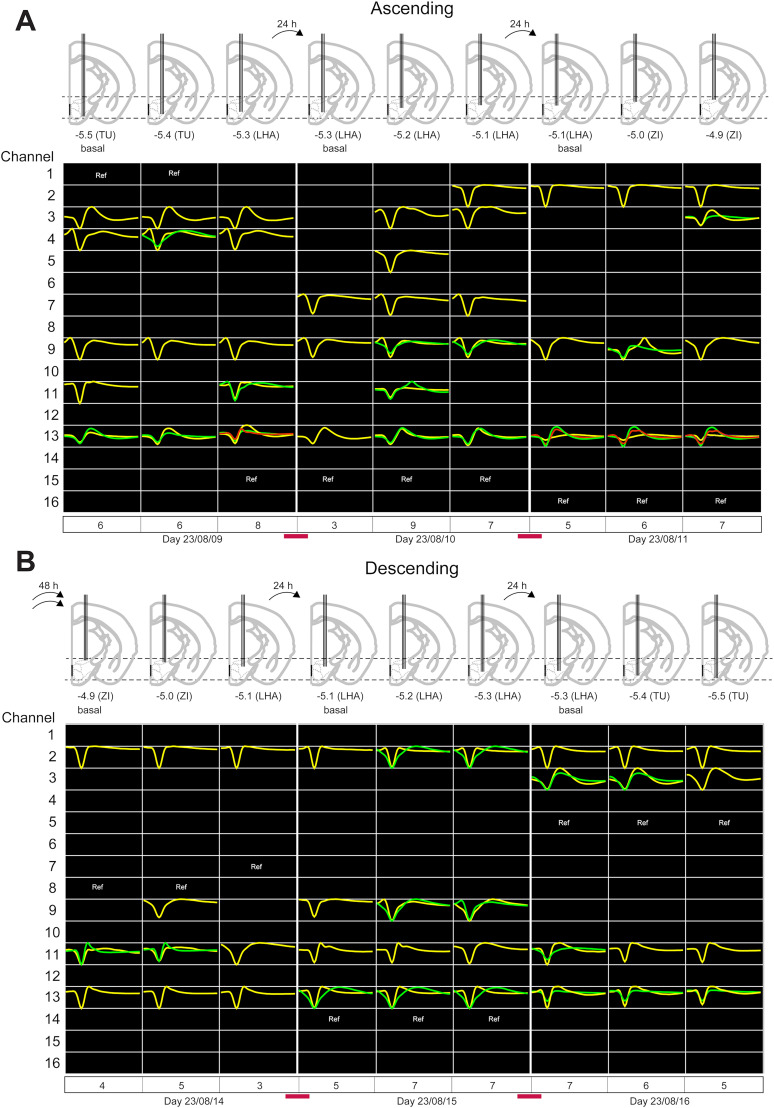
Representative extracellular recordings during sequential OptoDrive movements. ***A***, Ascending the positioning system and moving the OptoDrive: The top panel shows placement in coronal brain slides, representing the positioning of electrodes during three sequential sessions of OptoDrive movements. Movements traverse three distinct brain regions: the TU, the LHA, and the ZI. The bottom panel shows single-unit extracellular waveforms from 16 channels recorded via the same electrode array. Individual waveforms are color coded (yellow, green, and red), and each row represents the recordings from a single electrode channel. ***B***, Descending of OptoDrive. The 24 h transitions are represented by one arrow in the top panel and a solid red line in the bottom panel. Ref indicates the channel used as a digital reference.

The primary objective of this study was to evaluate the yield of newly recorded neurons obtained with the OptoDrive in each experiment. Figure 5*A* presents representative waveforms from the initial formal recording session (2023/08/09) in the TU. In channel 4, a new single unit appeared (green), whereas a unit previously detected in channel 11 was lost. Following two additional 60 µm upward steps of the electrodes within the same session, the green unit on channel 4 disappeared, two new units emerged on channel 11, and a new unit (red) appeared on channel 13. Channel 13 thus recorded three distinct units, representing the maximum number of single units isolated on a single channel during these experiments. Between recording sessions on different days, the electrodes remained in the position from the final recording of the previous day.

Contrary to expectations, distinct units were observed during the 24 h postrecording period despite maintaining the same electrode location. Notably, in channel 7, a new neuron emerged (Fig. 5*A*; see the red line between the dates 2023/08/09 and 2023/08/10). The next day, the electrodes were moved through the LHA, and 19 units were recorded for the entire session (Fig. 5*A*, 2023/08/10). Specifically, for the following 24 h postrecording, the units recorded in channels 3, 7, and 11 declined, passing from 7 to 5 units (Fig. 5*A*, transition between 2023/08/10 and 2023/08/11). The OptoDrive was advanced through the ZI region, resulting in an enhanced yield within channel 3 (see yellow and green waveforms), as evidenced by the emergence of two distinct single units (Fig. 5*A*, date: 2023/08/11).

#### Descending movement of the automatic OptoDrive

The next step was investigating whether recordings could continue across the same three regions during descending movements. Figure 5*B* depicts waveforms for three consecutive recording sessions while the OptoDrive descended. Notably, 48 h postrecording, while the OptoDrive remained in the ZI region, two new units appeared in channel 11 (green and yellow), and the unit observed in channel 9 disappeared (Fig. 5*A*, final panel on the right; Fig. 5*B*, the initial panel). A new single unit appeared at the first descending displacement when the electrodes passed through the ZI (channel 9). During the second movement, two units in channels 9 (yellow) and 11 (green) disappeared (Fig. 5*B*). On 2023/08/15, three novel units emerged during the OptoDrive's ventral descent through the LHA toward the TU regions. However, notably, during the LHA-TU transition, the two units initially present in channel 9 subsequently disappeared (Fig. 5*B*, between 2023/08/15 and 2023/08/16). Our findings indicate that recording novel units is feasible during intersession and intrasession periods, regardless of whether the electrode is ascending or descending. This suggests that any lesion caused by the electrode is moderate and allows continuous recording of neurons.

**Figure 6.**
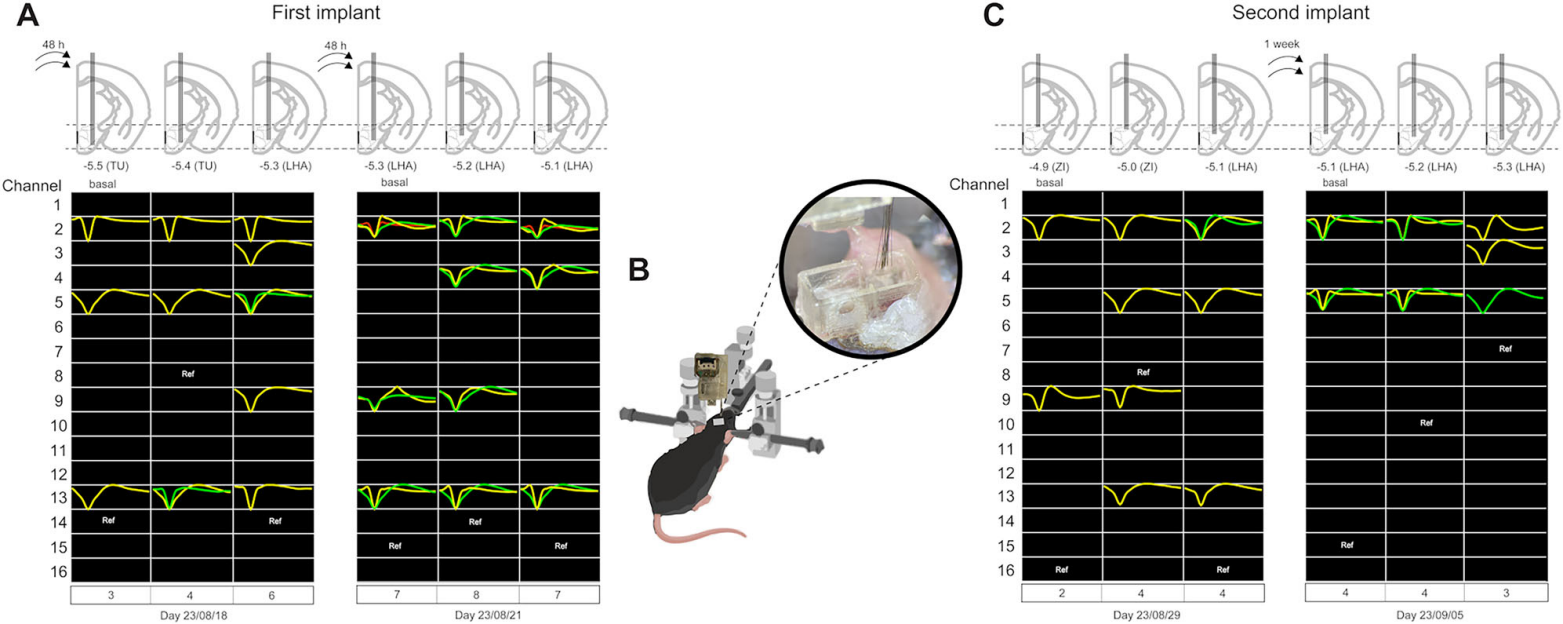
Representative extracellular recordings from the first OptoDrive implant and a second implant. ***A***, Representative final recording sessions for the initial OptoDrive implant are depicted. The top panel illustrates the positioning of electrodes during the final two sessions of OptoDrive electrode movements in coronal brain slides. The bottom panel presents single-unit extracellular waveforms from 16 channels recorded with the same electrode array. ***B***, Schematic representation of stereotaxic surgery for a subsequent OptoDrive electrode implant. ***C***, Two representative recording sessions for a second OptoDrive implant. The top panel depicts the placement of electrodes on coronal brain slides. The bottom panel displays single-unit extracellular waveforms from 16 channels recorded with the same electrode array. Individual waveforms are distinguished by color coding (yellow, green, and red), and each row corresponds to recordings from a particular electrode channel.

Given that brain tissue reactions such as glial formation and inflammation can disrupt recording sites, two supplementary sessions at 48 h intervals were used to assess changes in single-unit yield 2 weeks after the initial recording. Notably, three novel units were observed on channels 5 and 9, whereas two units—one from channel 11 and the other on channel 3—were no longer present (Fig. 5*B*, last right panel; Fig. 6*A*, the first panel). During the final recording session, the electrodes ascended through the LHA, resulting in the emergence of six novel units at channels 2, 4, and 9 (Fig. 6*A*, between dates 2023/08/18 and 2023/08/21). Our experimental results provide evidence of OptoDrive's ability to facilitate the recording of neuronal signals through three distinct brain regions over 13 d (from the first recording session on 2023/08/09 to the last recording of the first implant on 2023/08/21, which were 13 d apart).

#### Second implant

To counter potential electrode tip obstruction by glial cells or blood and signal sensitivity loss, a new OptoDrive was implanted in the same subject. Under isoflurane anesthesia, the original OptoDrive was unscrewed and easily extracted from the baseplate for replacement. Figure 6*B* illustrates the attachment of a replacement OptoDrive to the baseplate. The subject recovered in its home cage for 1 week.

Two consecutive recording sessions, 1 week apart, were subsequently conducted using the newly implanted OptoDrive. During microdrive advancement through the ZI, a new single unit emerged in channel 2, whereas a previously recorded single unit in Channel 9 was lost (Fig. 6*C*). Furthermore, while descending the OptoDrive through the LHA, a new single unit appeared for channel 3, but two units were absent (one at channel 2 and the other at channel 5). Notably, the single units recorded after the initial recording session, whereas the OptoDrive remained at the LHA, exhibited waveform changes compared with the beginning of the subsequent recording session. Notably, single units were still recorded between 2023/08/29 and 1 week later on 2023/09/05 (Fig. 6*C*). Thus, the first recording (August 9, 2023) to the last recording (September 5, 2023) of the second implant spanned 27 d of intermittent recordings in one mouse. OptoDrive supports multiweek signal stability. Its effortless reimplantation makes it valuable for studying the neural correlations among learning, memory, and diverse physiological processes.

#### OptoDrive recordings

**Figure 7.**
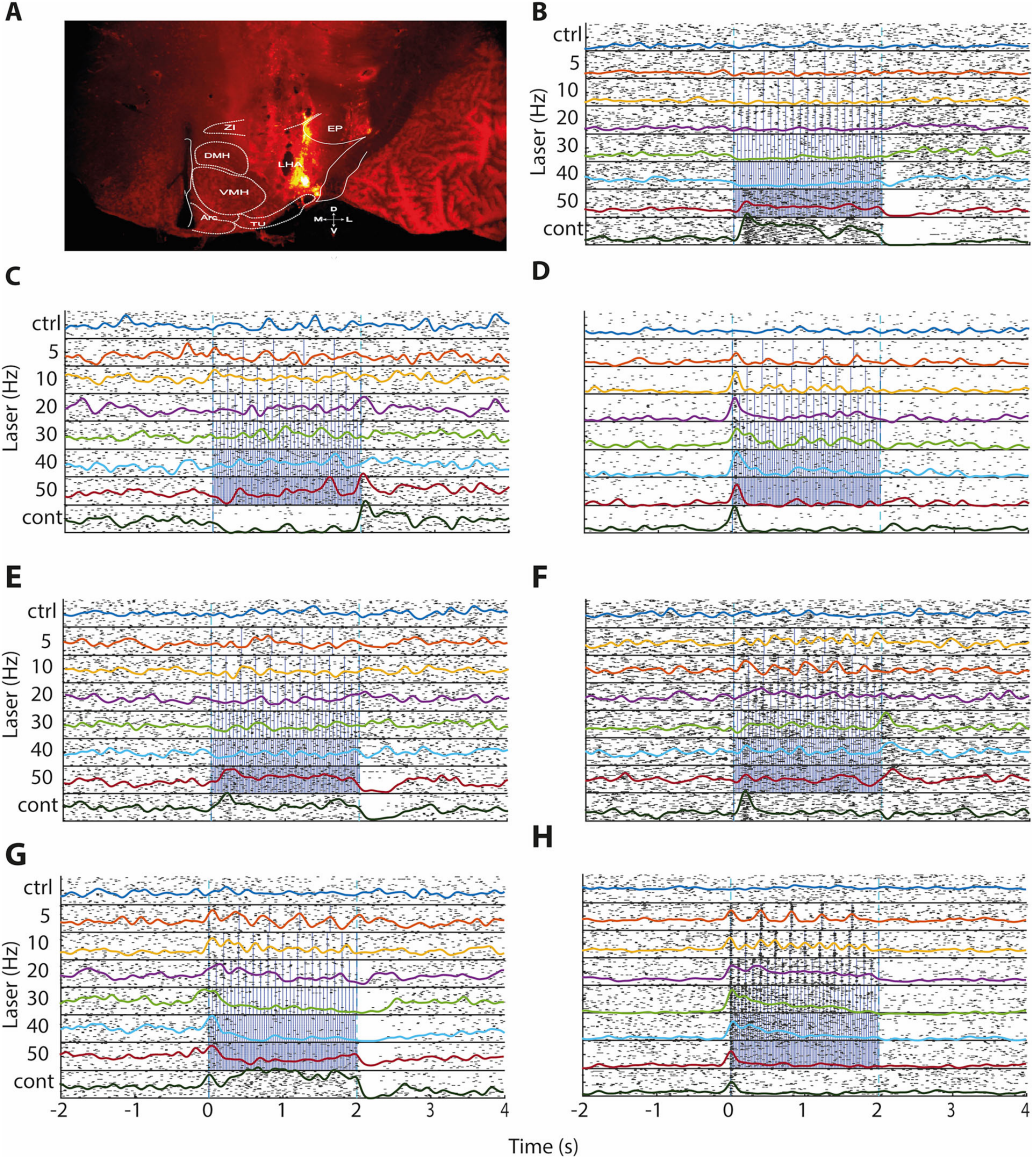
Optogenetic modulation of GtACR2-expressing neurons in the LH via OptoDrive. ***A***, Histological verification of OptoDrive placement in the ventral LH. The image shows the optical fiber track (indicated by the hole in the tissue) and the fluorescent track left by the electrodes. ***B–H***, Representative raster plots and overlapping peristimulus time histograms (PSTHs) of seven LH neurons expressing GtACR2, demonstrating diverse responses to optogenetic stimulation. Each panel shows neuronal activity during stimulation with blue light (473 nm) at various frequencies, presented in a randomized order: no stimulation (control; ctrl); 5, 10, 20, 30, 40, and 50 Hz; and continuous (cont) stimulation. Each black tick represents an action potential; blue ticks indicate laser pulses. Neuronal activity is aligned with the onset of the laser pulse (time = 0 s). Each recording session lasted for 30 min. ***B***, Neuron 1: This neuron exhibited a brief period of inhibition followed by activation during continuous stimulation. ***C***, Neuron 2: This neuron was inhibited during continuous stimulation. ***D***, Neuron 3: This neuron displayed a phasic increase in the firing rate at the onset of stimulation. ***E***, Neuron 4: This neuron showed increased activity during stimulation, followed by a period of inhibition after stimulation ceased. ***F***, Neuron 5 exhibited a delayed peak response at ∼200 ms postlaser onset (most likely a polysynaptic response). This peak was more prominent during continuous stimulation than at other laser frequencies. ***G***, Neuron 6: This neuron exhibited firing rate inhibition at 30, 40, and 50 Hz stimulation. Paradoxically, under continuous optostimulation, the firing rate of the same neuron increases. ***H***, Neuron 7: This neuron exhibits a phasic spike at each laser pulse, reliably following 5, 10, 20, and 30 Hz frequencies. Then, at 40 and 50 Hz, the neuron could not follow these frequencies, reliably exhibiting some jitter. During continuous laser irradiation, neurons, after a brief phasic response, exhibited robust inhibition. See Extended Data Figure 7-1 for other recordings made in the laboratory of the neurobiology of appetite, Cinvestav.

To validate the feasibility of recording extracellular neuronal activity during optogenetic manipulation via our microdrive (OptoDrive), we implanted it into the LHA of a Vglut2-IRES-cre mouse expressing stGtACR2, a light-gated anion channel (Mahn et al., 2018). Figure 7*A* depicts the histology of the mouse implanted with the OptoDrive (this time with a fiber optic). More tissue damage is caused by adding fiber optic material than by implanting the electrodes alone (Fig. 4). To mitigate this damage, the fiber optic is rigidly affixed to the OptoDrive main body, allowing only the electrodes to move independently.

10.1523/ENEURO.0015-25.2025.f7-1 Figure 7-1 Additional neurons recorded from other mice currently used in the laboratory neurobiology of appetite, Cinvestav. Download Figure 7-1, DOCX file.

Optogenetic stimulation over 5–50 Hz and continuous illumination elicited diverse neuronal responses, notably robust at 40–50 Hz and with constant light (Fig. 7). Figure 7*B* illustrates a neuron exhibiting a biphasic response: initial, transient inhibition followed by prolonged tonic activation lasting ∼2 s during stimulation. Conversely, Figure 7*C* shows a neuron with a sustained inhibitory response during continuous laser illumination. Figure 7*D* demonstrates a neuron that responded with a phasic increase in the firing rate at the onset of stimulation. Finally, Figure 7*E* shows a neuron displaying rapid phasic activation at 50 Hz and during continuous stimulation, followed by a poststimulus inhibitory rebound, a pattern reminiscent of the response in Figure 7*B*. The response properties of three additional neurons to various frequencies of optogenetic stimulation were investigated (Fig. 7*F–H*). Neuron 5 (Fig. 7*F*) displayed a delayed firing peak ∼200 ms poststimulus, indicative of a polysynaptic response; this peak's amplitude was significantly greater with continuous stimulation than with pulsed (5–50 Hz) stimulation. Neuron 6 (Fig. 7*G*) exhibited frequency-dependent firing rate modulation: pulsed stimulation at 30–50 Hz suppressed firing below baseline (no laser), whereas continuous stimulation significantly increased firing above baseline. Neuron 7 (Fig. 7*H*) showed a phasic response to low-frequency pulsed stimulation; at 5–30 Hz, each laser pulse reliably evoked a phase-locked single spike, although the jitter increased at higher frequencies (40–50 Hz). Continuous stimulation of Neuron 7 produced a brief initial phasic response. These results demonstrate that OptoDrive can reliably record optogenetically evoked neuronal activity in a freely moving mouse.

## Discussion

The OptoDrive, a novel, low-cost optogenetic system for chronic, long-term neural recordings in freely moving mice, was introduced. It features a detachable, reimplantable drive unit, an automated 16-channel tungsten-wire array (4 × 4, 15 µm resolution), and an integrated fixed optical fiber (200 µm, 0.39 NA) for stimulation, minimizing tissue damage as only the electrodes move. Future versions may add a 400 µm fiber for combined recording and fiber photometry. The OptoDrive costs ∼20USD (excluding EIB-16, pins, microwires, and fibers); most components are reusable, except for microwires and the baseplate. Its control system, which uses an A4988 Driver (∼2USD) and Arduino Uno R3 (∼23USD), is easily implemented. Compared with other systems, OptoDrive's stepper motor enhances user-friendliness, precision, and electrode stability, improving spatial localization. Future brain atlas integration for real-time electrode position estimation will further refine the targeting and streamline workflows. Proof-of-concept experiments demonstrated nearly month-long continuous LHA recordings and recording of evoked responses from optogenetic silencing (GtACR2) in a freely behaving mouse.

Our novel OptoDrive is well suited for long-term chronic recording and reimplantation in freely behaving mice. Our results revealed that single-unit waveforms are dynamic and stable, even when the electrodes were maintained at the same location. This finding agrees with our experience with fixed electrode arrays (Villavicencio et al., 2018). New neurons likely appeared spontaneously alongside stable ones, suggesting a dynamic yet stable recording environment. As expected, moving the OptoDrive introduced new signals, even in previously silent channels. Various GtACR2 responses demonstrate that the silencer's effectiveness is not uniform, depending on optogenetic parameters such as frequency. This highlights a known optogenetics issue in interconnected circuits: manipulating one cell type can activate or inhibit surrounding cells (Bernard, 2020). Postlaser shutoff activity rebound (positive/negative) was also observed, suggesting that neurons attempt spiking homeostasis, as previously reported in mice with fixed electrodes (Prado et al., 2016; Luis-Islas et al., 2022).

The implementation of motorized systems for extracellular recordings is often challenged by cost, control systems, technology, and reusability. These issues are evident across several designs (Fee and Leonardo, 2001; Yamamoto and Wilson, 2008; Caballero-Ruiz et al., 2014; Dabrowski et al., 2015). For example, a novel micropositioning system for 16 tetrodes (64 channels), where a single piezo actuator (PK3CMP2, $65) is employed in an inchworm configuration with a temperature-controlled PCM, was reported (Smith et al., 2022). A weight of 4.5 g and dimensions of 25 × 15 × 31 mm are noted. Although an excellent alternative for recordings in unrestrained small animals is represented by the system from Smith et al. (2022), widespread adoption may be hindered by potentially complicated fabrication and control, underscoring the ongoing search for optimal solutions. Previous works reported screw-driven microdrives with attached optical fiber (Anikeeva et al., 2012; Freedman et al., 2016; Osanai et al., 2019). Anikeeva et al. (2012) reported the “Optetrode,” which consists of an optical fiber and tetrodes; the system can perform displacements with a resolution of 454 μm per turn, weighing 2 g. Freedman et al. (2016) reported the OptoZIF Drive, which uses a ZIF connector and four independent mobile electrode recordings (200 μm resolution per turn) and a fixed optical fiber (∼3 g). Osanai et al. (2019) presented a hybrid multiregion system with a mobile optical fiber coupled to a silicon probe for one region via a screw with a pitch of 300 µm and nine independent mobile tetrodes for the other region via a 160 µm pitch custom screw; the total weight of this system is 5.9 g. Traditional screw-driven mechanisms in microdrives, limited to ∼40–75 μm resolution, are functional, inherently risk electrode damage due to manual manipulation and lack precision for demanding spatial control, and they present issues related to backlash and stability.

### Limitations of the study

A key limitation is movement-restricting cables; a wireless system would enhance mobility and welfare. Future OptoDrive iterations should also consider replacing tungsten wires with silicon probes (e.g., Neuropixels) to increase neuronal yield and improve recording site localization significantly.

### Conclusion

OptoDrive, a novel, low-cost, lightweight microdrive for chronic neural recordings in freely moving mice, is introduced. It overcomes limitations such as short recording durations and the absence of integrated optogenetics by enabling long-term, reimplantable recordings with concurrent optogenetic manipulation. Its detachable, automated drive ensures stable, extended recordings (e.g., nearly month-long LHA data). An integrated optical fiber facilitates simultaneous optogenetics, as shown by GtACR2-evoked responses and is currently used as a laboratory recording workforce (Extended Data Fig. 7-1). OptoDrive is a robust, versatile solution for long-term brain activity and behavior studies in freely moving mice.

## Synthesis

Reviewing Editor: Mark Laubach, American University Decisions are customarily a result of the Reviewing Editor and the peer reviewers coming together and discussing their recommendations until a consensus is reached. When revisions are invited, a fact-based synthesis statement explaining their decision and outlining what is needed to prepare a revision will be listed below. The following reviewer(s) agreed to reveal their identity: Tim Schroeder.

Thank you for sending your work to eNeuro! Your paper was reviewed by two experts and their comments are given below in full. Please revise accordingly to address all concerns that were raised.

Reviewer 1

The overall goal of this manuscript is to create an updated, light-weight Microdrive design called Optodrive that is suitable for mice (as opposed to a previous design published specifically for freely moving rats which used an automated motor as well). Additionally, the authors added capability for optogenetic manipulation by including an optic fiber to the design. They have improved the previous design by maintaining a low-cost option to build a light-weight optodrive intended specifically for freely moving mice, which is also run by an automated motor drive. The main novelty of this manuscript is 1) the addition of the optic fiber to the Microdrive to create the Optodrive and 2) lightening the total weight of the microdrive to be suitable for mice. I would suggest emphasizing this upfront and reframing the manuscript to focus on these main additions and how this improves previously published manuscripts.

Major:

-Flow: I would suggest putting fabrication and assembly into the methods section of the paper, such that it can read more as a protocol for others to potentially build and use this, expanding its use to the audience. See other eNeuro "novel tools and methods" manuscripts for inspiration, such as Silva et al., 2024 or Quansah Amissah et al., 2023. Then, the results can just be proof-of-concept emphasizing stable recordings, opto perturbation, and measurement quantification, and the methods will hold the bulk of the fabrication / assembly of the device.

- Technical clarifications: Is there a link for the PCB parts? A bill of materials including links for parts, along with 3d printed files (.stl) should be included. If the goal is for someone to re-create and use this device, they will need to know exact screw sizes, pin sizes, where to purchase EIBs, wire, what material it is 3d printed out of to make it a lighter material for mice, etc.

-Are the electrodes housed in polyimide tubing or are they free wires moving through a metal cannula guide tube? If they are "free wires", how can you ensure they are not bending or splaying during implant or ascending/descending the Microdrive? Typically, the polyimide tubing provides a bit of a scaffold for the wires, or alternatively wires are glued in a manner around the optic fiber to ensure support / scaffold from the fiber. Is this the case for this setup? If so, please clarify how the wires are still maintaining their structure while being implanted and not splaying. Additionally, if they are simply glued to the optic fiber, does this affect the ability of the motor to advance the bundle up and down?

-There should be more detail emphasizing the optic fiber inclusion to the Microdrive. Is the optic fiber 200 or 400 um, and can both fit with the current setup? What is the minimum and maximum length required for the optic fiber to be? Are you constructing the optic fiber/ferrules together yourself, or purchasing it from a company? What length is the optic fiber above the end of the electrodes? Does the animal now have two cables (one to the EIB for recording, and one optic fiber? attached to them, and if so how is this done to prevent cable tightening / bundling or cable biting, since this is a freely moving animal? Is there any dustcap for the optic fiber, is there any wall/ cap/ protection for the freely moving mouse to not crack off the optic fiber's ferrule?

-I am still concerned about moving the electrode in the upward direction and still getting useful recordings and not creating damage, along with the stability of recordings across multiple implantations in and out each day, especially considering the optic fiber moving up and down each time. Typically, any movement in the upward direction is limited, especially due to potential damage to those neurons; given that an optic fiber is typically 200-400 um, this is a lot of potential damage to continue to increase and decrease the depth. Can you show any evidence for where the actual electrodes ended up (i.e., apply an electrolytic lesion to the pin on the EIB for a few channels to at least deduce the exact end position of the electrode), or an image of the brain post-mortem showing minimal damage? Additionally, is the brain shown in Figure 5 with only lowered electrodes in DiI or did this also have the optic fiber? I am surprised to not see any implant damage / evidence of the fiber track specifically here, and given that the major novel and new aspect of this manuscript is with the new inclusion of the optic fiber, I would suggest showing an image where it is clear that the optic fiber was also implanted

Minor:

1.I understand that the authors had mentioned some of the history of microdrives etc in the discussion, though I would suggest citing a few of these seminal studies in your introduction that have published probes for chronic recordings in freely moving animals (potential studies to include upfront: van Daal et al., Nature protocols, 2021 which is the updated protocol for neuropixels probes that has an updated ZIF connector; van Daal et al., Journal of Neural Engineering, 2020; Osanai et al., J Vis exp, 2019; Yamamoto and Wilson, JNP, 2008; Fee and Leonardo, 2001). I would also add in any references comparing with other optodrive or optrodes that exist (for either acute or chronic recordings), since this is the major point of the study. Additionally, a note in the discussion comparing with other optrodes or opto-inclusive microdrives would be nice, since this aspect is not so novel in head-fixed setups.

2.Abstract - I would caution against opening the manuscript by saying that extracellular recordings in freely moving mice require microdrives; one can use chronic fixed position electrode arrays to perform extracellular recordings. The benefit of microdrives is to (in theory, with a high degree of confidence) ensure you can record from a new population of neurons each day as you travel ventrally.

3.Lines 431-461 in the discussion are confusing and have some typos, and it is difficult to track which studies are being referred to. E.g., I believe it should read "Sato et al (2007) presented..." ; "Yamamoto and Wilson (2008) reported ..."; "Smith et al. (2022) reported"

4.Line 474: I believe you mean the EIB is purchased from Neuralynx?

5.I am still confused about the implant and the baseplate, and what parts are permanently attached onto the animal. I think some clarity on the baseplate upfront would be useful, i.e. explaining that the baseplate is the part that is glued and permanently affixed to the mouse's skull, but the rest of the implant can be removed and replaced.

6. Some clarity upfront is warranted on why the motorized Microdrive is more ideal than a traditional screw mechanism (e.g., screw one full turn of a screw to advance 75 um). Is the rationale that the motor makes this more human-user friendly for freely moving animals? A reminder in the text would be ideal for why the motor is being used in the first place since some people may argue this just adds weight to the implant.

Reviewer 2

The work presented in the manuscript has the potential to advance the field: The manuscript presents the design of a low-cost motorized microdrive for simultaneous recording with up to 16 wire electrodes and integration of a fiber for optogenetic stimulation. While the fundamental idea of the design is not novel, the acceptable (albeit very high for freely moving animals) weight of the implant and use of widely available low-cost components make it a potentially interesting alternative for cases in which the cost of other systems is prohibitive.

However, multiple details in the actual description of the work severely hinder that advancement:

(1) Replicability: While the authors provide a list of commercially available components, they do not share design files of the custom components or provide a license that allows replication. While the figures in the manuscript would, in theory, allow replication of the custom parts in the design, the need to replicate custom components from scratch effectively reduces the advancement of the field to the characterization of the positioning error of the commercially available stepper motor. However, as the authors do not mention the model of the stepper motor, this advancement is not practically useable. This sentiment extends through the whole manuscript: for example, the authors mention that the control system is based on an Arduino and A4988 driver, but give no details with respect to the actual implementation in their system.

(2) Related, cost: the authors present the microdrive as a low-cost solution (< 20 USD). However, while they analyze the cost of competing drives, they do not provide an overview of the cost associated with individual parts, especially custom pieces. For example, the mentioned 3D printer (Stratasys Connex 3) is a commercial high-end machine with a likely price tag of over 200 USD.

(3) The authors do not give sufficient information on the animals used in this study. The weight of the microdrive (~3.2g for the components, likely not including the weight of adhesive cement and acrylic. While the video supplied in the extended data, showing a healthy mouse with natural movement and rearing the home cage, suggests that the implant is sufficiently light, better description of the weight in relation to the weight of the mice would be useful.

To sum up, the microdrive presented in the article has the potential to advance the field, but the omission of crucial details in the current version of the manuscript precludes this advancement.

Generally, the work is described inconsistently and contains multiple claims that are not supported by the data presented: For example, the authors claim the Optodrive is a solution for long-term experiments (line 17/18: "we demonstrate stable recordings (...) for over a month" and line 509: "recordings (...) lasting up to 2 months"). However, the experimental data presented shows recordings for a 13 day period (e.g., line 364). Furthermore, the introduction and actual experiments describe a movement resolution of 60µm, while the discussion (line 471) claims 15µm. From initial description and presented data, it looks like three recording sessions were done per day, while in line 302-306, it is claimed that no more than two recordings were done per day. The additional conclusion of the same paragraph that as a consequence of considering the two (three?) recording sessions per day, the stability across days could be measured, is confusing at best. As mentioned below, the inter-recording interval of 10 minutes is on the short side, raising doubts whether these sessions can actually be regarded as independent.

Additionally, "stability" is not at all defined in the manuscript. From the text, it can be inferred that it is the ability to record putative single-unit activity, which would be mainly influenced by inflammatory activity and gliosis. As the diameter of the wire used as electrodes is not communicated, it is difficult to put this finding into perspective. No information is given about long-term stability of the implant when it includes an optical fiber. When describing the putative single units recorded in the first experiment, the authors might also imply that long-term recording of individual, defined units is possible. (This is not entirely clear, and not explicitly stated.) Depending on thickness of the wire electrodes and their density when penetrating the brain, moving the electrode array might also (temporarily) displace neural tissue by pulling it in the same direction as the electrodes. It can take significantly longer than the 10 minute inter-recording interval used here for the brain tissue to settle, possibly resulting in spurious "stable" recordings within the same day.

The manuscript contains data from n=2 animals, carrying implants with and without optical fiber, respectively. Given that the authors claim their "lab currently uses it as the recording workforce", I would have expected the manuscript to include more extensive statistics and data of a larger group of animals regarding neuronal yield. If additional data from other experiments in the lab is available, presenting more extensive statistics on neuronal yield, recording longevity, and how they potentially change with effect of re-implantation, would be useful for readers to evaluate the performance of the Optodrive and the claim that it "increases the success of electrophysiologial studies in freely moving mice" (line 71). This would also allow to better compare the Optodrive to other microdrives in terms of long-term stability of the implant and recording quality.

While the manuscript claims long-term recording stability and does not discuss this aspect, it looks like the cortical areas above the recording side occurred visible deformation (there is a bit dent in the left hemisphere in Figure 4 above the marked electrode track). For the reader, it would be great to explain this deformation and its possible cause (for example, is this due to implantation of the base, due to reimplantation, or does it always happen) in order to make an informed decision on the suitability of the Optodrive for other recording locations.

In terms of animal welfare and behavior: the video shows a healthy looking mouse with normal home cage movements, which is great. As the Optodrive is on the heavy side for implants in freely moving mice (3.2g without the weight of the cement/acrylic used in the surgery), a more thorough quantification of "normal movement", for example by video analysis of implanted and unimplanted animals, would have been great. In addition, the text does not mention the weight of the mice used, making it difficult to put the movement seen in the video into context.

A side note for welfare: the manuscript does not mention any kind of analgesia, neither before, during, or after surgery. Was no analgesia applied, and is this in line with institutional welfare protocols in the author's institution?

Regarding the characterization of the Optodrive, it is not clear why the 60µm resolution was chosen. For readers, it would have been useful to also validate the placement error and movement for other step sizes in case specific applications required different step sizes or lower placement variability. Connected to this aspect, the benefits of having a motorized microdrive in this constellation could be made clearer - my assumption is that the benefits of being able to repeatedly and move the electrodes precisely, even when the animal is not restrained by the experimenter can outweigh the disadvantages (~30-50% extra weight due to motor and the necessity to connect an extra cable to drive the linear actuator). However, from the current manuscript it is not clear whether these are the actual benefits of the Optodrive.

The manuscript would benefit from additional proofreading and editing to improve structure and clarity. For example:

- Line 43: "neuropixel technology" should probably be "silicon probe technology", as Neuropixels probes are just one (albeit the now most prominent) type of silicon probes

- Line 62/64: "the system's automated features, such as (...) attachment of a fiber optic". Attachment of an optic fiber is not an automated feature

- Mix between active voice (e.g. line 140) and passive voice (most of the manuscript)

- Line 148 refers to the mouse in singular, while two mice were used

- Figure 2 (g) should probably read "assembly of sections (e) and (f)" instead of "assembly of sections (d) and (f)"

- Line 352 "Ref means a common average channel". Unclear, as usually, common average references are re-referenced signals based on the common average of multiple channels, not a specific physical reference channel.

- Line 356: while gliosis and inflammation can probably affect the electrical characteristics of neurons, in this context is probably more likely the influence of those on the electrodes and the recording of the electrical response of the neurons is meant

- Line 357: session were claimed to be conducted to "assess the longevity of signal noise". Analyses only show assessment of putative unit count, no signal noise assessments

- Line 416 "opto-simulated" instead of "opto-stimulated"

- Placement of references in the discussion. Here, for most (but not all) references the punctuation is off (for example in line 436 "(Yamamoto and Wilson). Reported" instead of ".(Yamamoto and Wilson) reported".

- Line 474 "Neuralix" instead of "NeuraLynx"

The manuscript claims the Optodrive to be a cost effective solution and provides instructions to assemble the Optodrive. However, the exact materials used are not specified. For example, the low-cost motor employed is not characterized, making it impossible to validate the claim that it is more affordable than the devices used in previous designs. Other crucial components, such as the motor driver, are not described either. Custom parts which (e.g., 3D printed parts) are not provided, and the use of a high-end industrial 3D printer is described (The Stratasys Connex 3 used is a commercial high-end machine with a likely price tag of over 200 USD). This makes it difficult to assess the actual costs for labs to recreate the Optodrive. As the authors neither publish the design files nor explicitly offer to share them, recreating the Optodrive from the manuscript would therefore essentially require reverse engineering from the incomplete descriptions and figures, severely limiting its use to the broader community. While this can be a conscious decision, for example to guard intellectual property, it makes the benefit of the Optodrive to be low-cost less relevant to the reader.

All in all, I do think that the Optodrive is a well-engineered microdrive that has the merit to be published in eNeuro, and while it looks like the work does contain the elements needed, at least the gaps in the manuscript listed above will need to be addressed. This should not require additional (animal) experiments, if more data is available in the lab. If it is feasible to conduct additional measurements to characterize the positioning error for additional step sizes, this would give other potential users of the Optodrive a better way of judging whether the microdrive is suitable for their application.
