# Supplementary material for: Automatic OptoDrive for Extracellular Recordings and Optogenetic Stimulation in Freely Moving Mice
Source: eNeuro. 2025 Jun 18;12(6):ENEURO.0015-25.2025. doi: 10.1523/ENEURO.0015-25.2025 (PMC12203764; doi:10.1523/ENEURO.0015-25.2025)
Supplement: Figure 2-1 — Optodrive Bill of Materials. Download Figure 2-1, DOCX file. [file eneuro-12-ENEURO.0015-25.2025-s002.docx]

| OptoDrive Bill of materials | | | | | | | |
| --- | --- | --- | --- | --- | --- | --- | --- |
| Item | Description | Image | Quantity required | unit price USD | Webpage seller | Weight [g] |  |
| 1 | Miniature linear actuator | 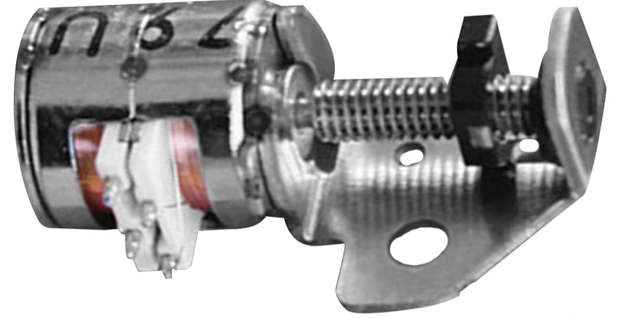 | 1 | $ 0.77 | <https://rb.gy/cl972t>  (Accessed 11-03-2025) | 1.1686 |  |
| 2 | 4p FPC/FFC 0.5mm pitch connector | 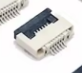 | 1 | $ 0.46 | <https://rb.gy/sil2kh>  (Accessed 11-03-2025) | 0.1064 |  |
| 3 | Miniature linear actuator PBC (Thickness 0.6 mm, hole size 0.3mm) | 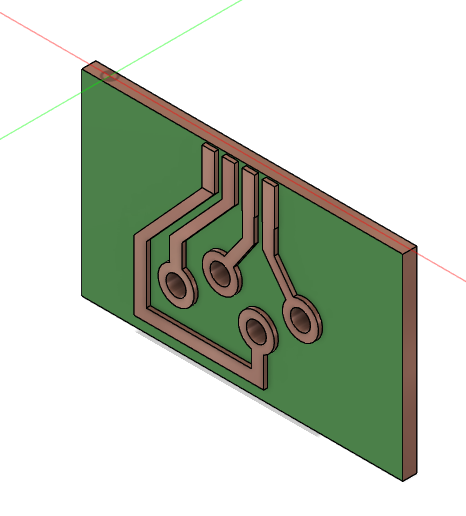 | 1 | ~$ 0.65 | <https://www.pcbway.com/>  0’’(Accessed 11-03-2025)  PCB files are includes | 0.075 |  |
| 4 | Electrode shuttle | 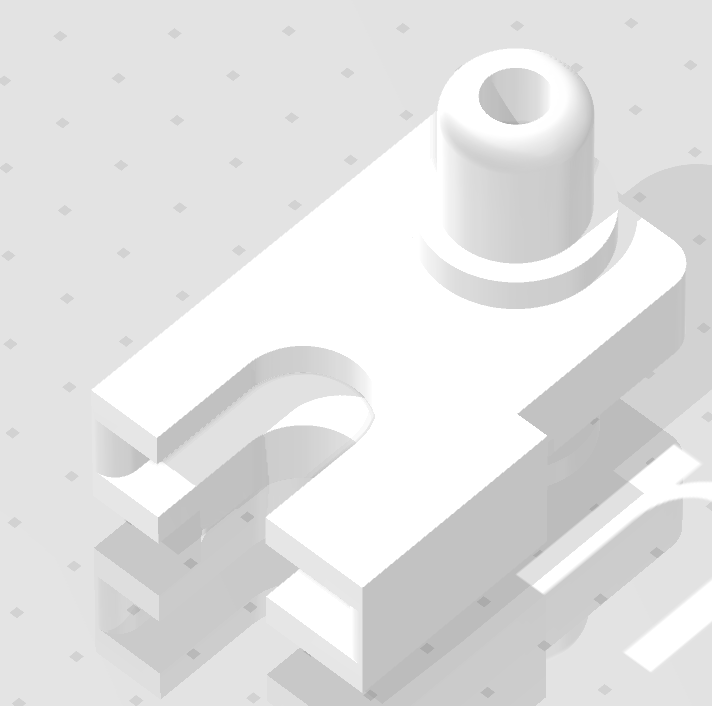 | 1 | ~$ 4 | STL file Included | 0.02315 |  |
| 5 | Compression spring (0.2mm Wire diameter, 5mm Length, 3.0mm Outer diameter | 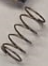 | 1 | $ 0.16 | <https://rb.gy/abf2yp>  (Accessed 11-03-2025) | 0.0129 |  |
| 6 | Support board | 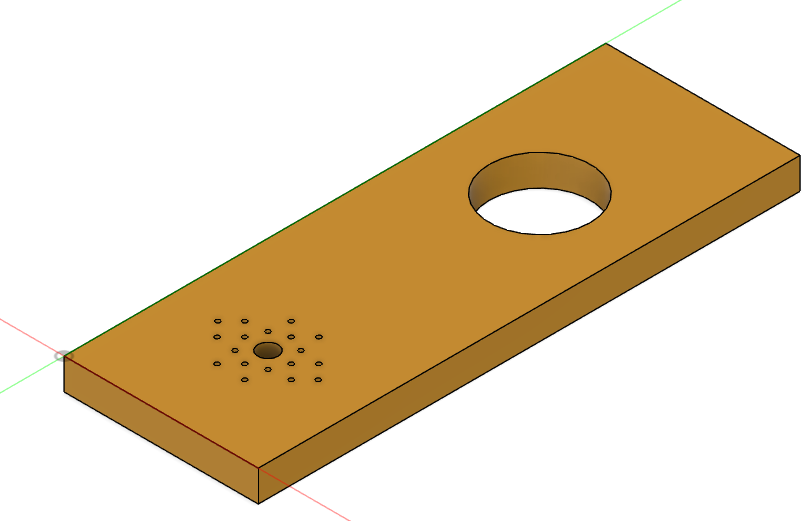 | 2 | ~$ 2 | IGES file Included | 0.0144 |  |
| 7 | **B**ushing 2 mm length (Cut from G18 cannula) | 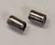 | 2 | ~$ 0.2 | <https://acortar.link/bXOpxH> (Accessed 11-03-2025) | 0.0129 |  |
| 8 | Guide 15 mm length (Cut from G20 cannula) | 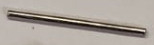 | 1 | ~$ 0.2 | <https://acortar.link/yywCNM>  (Accessed 11-03-2025) | 0.0464 |  |
| 9 | Base plate | 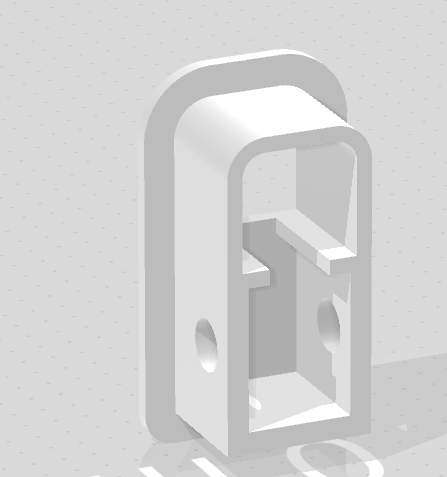 | 1 | ~$4 | STL file Included |  |  |
| 10 | OptoDrive main body | 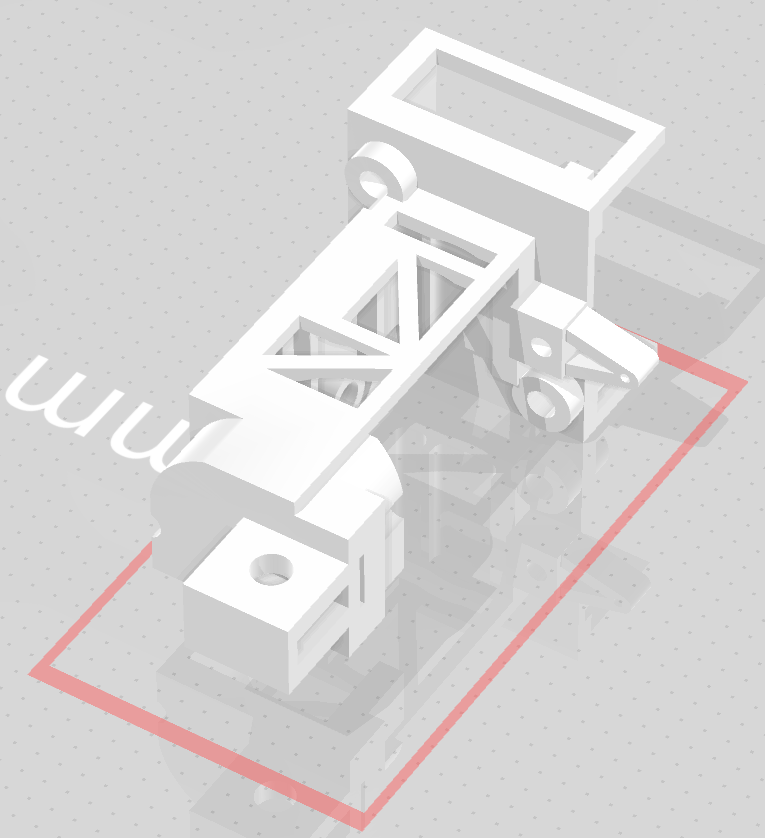 | 1 | ~$4 | STL file Included | 0.5702 |  |
| 11 | Tungsten microelectrodes ( 35 μm diameter formvar insulated ) | 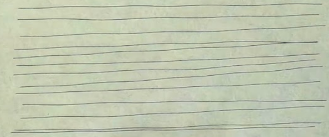 | 16 | N/A | <https://rb.gy/7kec9a> (Accessed 11-03-2025) | 0.0192 |  |
| 12 | Optical fiber with its ferrule | 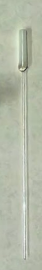 | 1 | N/A | <https://www.thorlabs.com/thorproduct.cfm?partnumber=FT200UMT>  (Accessed 11-03-2025) | 0.0483 |  |
| 13 | Gold-plated electronic interface board EIB-16 | 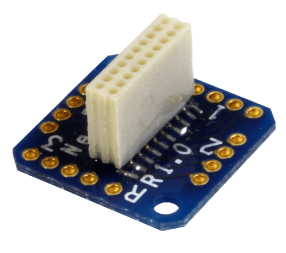 | 1 | N/A | <https://neuralynx.fh-co.com/research-hardware/animal-interfaces/eibs/eib-16/>  (Accessed 11-03-2025) | 0.2407 |  |
| 14 | Gold plate pins | 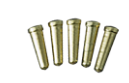 | 16 | N/A | <https://neuralynx.fh-co.com/research-hardware/animal-interfaces/microdrive-preparation/large-eib-pins/> | 0.0770 |  |
| 15 | Fixing nut | 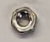 | 1 | $ 0.06 | <https://rb.gy/yyrjih>  (Accessed 11-03-2025) | 0.0577 |  |
| 16 | Fixing screw | 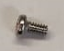 | 1 | $0.05 | <https://rb.gy/iga0gu>  (Accessed 11-03-2025) | 0.0724 |  |
| 17 | Screws | 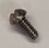 | 2 | $0.1 | <https://rb.gy/fo7f9g>  (Accessed 11-03-2025) | 0.1162 |  |
| 18 | Body cover | 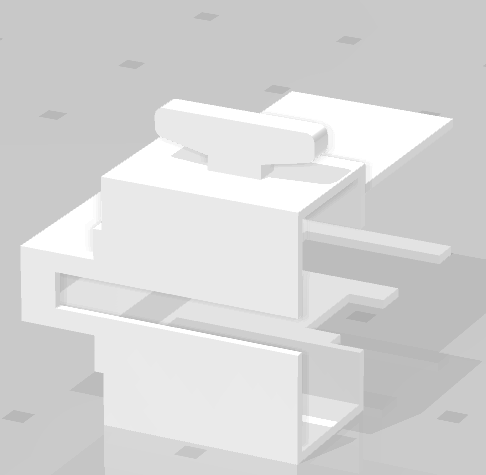 | 1 | $4 | STL file Included | 0.5897 |  |
| 19 | Stepper motor driver A4988 | 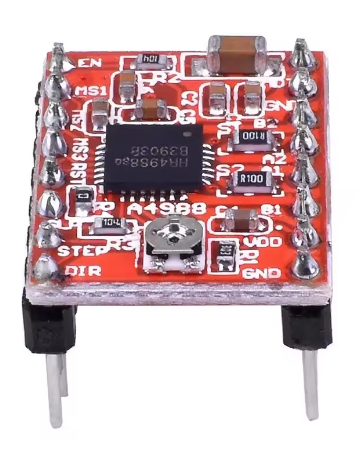 | 1 | $2 | <https://rb.gy/iga0gu> (Accessed 11-03-2025) |  |  |
| 20 | .95Arduino UNO R3 | 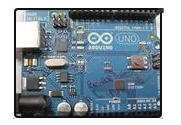 |  | $23 | <https://rb.gy/y81wmz> (Accessed 11-03-2025) |  |  |
