# Supplementary material for: Automatic OptoDrive for Extracellular Recordings and Optogenetic Stimulation in Freely Moving Mice
Source: eNeuro. 2025 Jun 18;12(6):ENEURO.0015-25.2025. doi: 10.1523/ENEURO.0015-25.2025 (PMC12203764; doi:10.1523/ENEURO.0015-25.2025)
Supplement: Figure 7-1 — Additional neurons recorded from other mice currently used in the laboratory neurobiology of appetite, Cinvestav. Download Figure 7-1, DOCX file. [file eneuro-12-ENEURO.0015-25.2025-s003.docx]

| **Extended Data Fig. 7-1**. Additional neurons recorded from other mice currently used in the laboratory neurobiology of appetite, Cinvestav. | | | |
| --- | --- | --- | --- |
| Mice name | Sessions | Total neurons | Time implant |
| EM6 | 4 | 6 | 5 days |
| EM13 | 17 | 30 | 34 days |
| EM5 | 2 | 4 | 2 days |
| M9T | 2 | 2 | 2 days |
| M4T | 7 | 7 | 12 days |
| Earch2 | 15 | 23 | 36 days |
